# Interindividual Brain and Behavior Differences in Adaptation to Unexpected Uncertainty

**DOI:** 10.3390/biology12101323

**Published:** 2023-10-10

**Authors:** Célia Soussi, Sylvie Berthoz, Valentine Chirokoff, Sandra Chanraud

**Affiliations:** 1 INCIA CNRS 5287, University of Bordeaux, 33076 Bordeaux, France; soussi@cyceron.fr (C.S.); valentine.chirokoff@ephe.sorbonne.fr (V.C.); sandra.chanraud@u-bordeaux.fr (S.C.); 2 UNICAEN, INSERM, U1237, PhIND “Physiopathology and Imaging of Neurological Disorders”, NeuroPresage Team, Cyceron, Normandy University, 14000 Caen, France; 3 Department of Psychiatry for Adolescents and Young Adults, Institut Mutualiste Montsouris, 75014 Paris, France; 4 Ecole Pratique des Hautes Etudes, Section of Life and Earth Sciences, PSL Research University, 75014 Paris, France

**Keywords:** adaptation, exploration/exploitation trade-off, resting-state functional connectivity, associative learning, interindividual variability

## Abstract

**Simple Summary:**

To adapt to a new environment, people must choose between using strategies that previously produced the expected effect in a similar context and trying new strategies with no certainty of achieving the expected effect. This is known as the exploration/exploitation trade-off. Its underlying brain mechanisms and factors accounting for interindividual differences have been overlooked. Resting-state functional connectivity highlights the synchronicity of different brain regions at rest, i.e., basal or intrinsic connectivity. Here, we investigated the evolution of this synchronicity before/after a task involving environmental uncertainty. To study the trade-off, we introduced uncertainty and cheated participants by introducing false feedbacks during an associative learning task. We examined the associations among the participants’ behaviors, changes in the brain intrinsic functional connectivity, and psychological factors. The sensitivity to uncertainty was used to create two groups. The groups displayed different patterns of evolution of regional connectivity from before to after the task. We also found a trend whereby higher scores of anxiety and greater propensity to doubt about actions were positively linked to these patterns. These results provide additional arguments in favor of short-term plasticity in basal brain connectivity and clues about the interindividual factors influencing neural and behavioral adaptation.

**Abstract:**

To adapt to a new environment, individuals must alternate between exploiting previously learned “action–consequence” combinations and exploring new actions for which the consequences are unknown: they face an exploration/exploitation trade-off. The neural substrates of these behaviors and the factors that may relate to the interindividual variability in their expression remain overlooked, in particular when considering neural connectivity patterns. Here, to trigger environmental uncertainty, false feedbacks were introduced in the second phase of an associative learning task. Indices reflecting exploitation and cost of uncertainty were computed. Changes in the intrinsic connectivity were determined using resting-state functional connectivity (rFC) analyses before and after performing the “cheated” phase of the task in the MRI. We explored their links with behavioral and psychological factors. Dispersion in the participants’ cost of uncertainty was used to categorize two groups. These groups showed different patterns of rFC changes. Moreover, in the overall sample, exploitation was correlated with rFC changes between (1) the anterior cingulate cortex and the cerebellum region 3, and (2) the left frontal inferior gyrus (orbital part) and the right frontal inferior gyrus (triangular part). Anxiety and doubt about action propensity were weakly correlated with some rFC changes. These results demonstrate that the exploration/exploitation trade-off involves the modulation of cortico-cerebellar intrinsic connectivity.

## 1. Introduction

Adaptation can be defined as the way individuals adjust their behaviors to fit environmental contingencies. To do so, one must not only learn which actions are efficient in specific situations and consolidate the learned associations between situations and actions but also be sensitive to the challenging fluctuations of the intrinsic and extrinsic environmental demands to navigate them adequately and flexibly. It is, therefore, necessary to alternate between (1) exploiting known action–outcome contingencies and (2) exploring other actions to gain knowledge about the environment, a process that has been referred to as the exploration/exploitation trade-off [1]. In other words, one has to evaluate environmental uncertainty to estimate whether or not there is a need for exploring and updating previously learned models [2]. As for uncertainty, it can be considered either as “expected” if the absence of the awaited outcome can be explained by already acquired knowledge on such fluctuations of the environment, or it can be defined as “unexpected” if an event fundamentally challenges the learned action–outcome contingencies [3].

Reinforcement learning problems enable studying the exploration/exploitation trade-off. The multi-armed bandit problem is the most used paradigm. Participants have to discover gradually which choice to make for maximizing gains amongst several options (i.e., arm) with various unknown probabilities a priori for each one to win [4]. This paradigm characterizes how the participants’ decisions fit the problem and evolve over time based on the acquisition of new information [5]. Different versions of this type of task, with various numbers of arms, exist in the literature [6,7,8].

Another type of paradigm is the so-called clock task [9], where participants have to discover gradually when to stop a running clock before a whole turn of the clock hand for maximizing gains. Different rewards that are not known a priori correspond to the time at which the clock is stopped, and participants can gain information about the different rewards attributed to each area of the clock across trials.

Finally, paradigms referred to as an “observe and bet” task have been used to investigate each of the two components of the exploration/exploitation trade-off separately. In each trial, the participants can either observe a draw—an action that is known a priori to be associated with no gain or cost—or make a choice, and thus bet on the expected value of the choice based on the information gathered progressively. For example, participants can be informed that a deck of red and black cards contains more cards of a certain color, with either more red than black cards or the opposite. The participants have three possible choices: bet on red, bet on black, or observe which card is drawn to obtain more information on the composition of the deck, thereby maximizing gains [10]. Others have used a version with two lights and the instruction to predict which of either the blue or the red one would light up [11].

While the exploration/exploitation trade-off has been widely investigated in both humans and animals at the behavioral level [12], very few studies have further evaluated the potential influence of personality traits on this trade-off when facing unexpected uncertainty [13], although there may be important interindividual variability in the sensitivity to uncertainty [14]. Moreover, poor ability to balance between exploring and exploiting has been reported in various mental disorders (e.g., schizophrenia [15], addiction [9], and anxiety [16]). From a clinical perspective, being able to characterize individuals’ adaptation to unexpected environmental changes with behavioral paradigms estimating this trade-off could be of great interest [17]. To date, too little is known to guide clinical practice, and the underlying mechanisms of both behavioral and brain interindividual variability involved in the trade-off are yet to be fully understood in healthy populations [12]. One source of such variability in estimating and reacting to uncertainty could be the influence of personality traits [18].

At the cerebral level, studies using task-based functional magnetic resonance imaging (fMRI) have identified distinct neural correlates for exploration and exploitation behaviors. Exploitation has been linked to activity in the ventromedial prefrontal cortex [6,7,11], orbitofrontal cortex [7], and the insula [11], suggesting that exploitative behaviors would rely on the brain areas that are part of the reward system [8]. Exploration has been linked to activity in the frontopolar cortex and intraparietal sulcus [5,19] and was suggested to be sustained by the attentional system [7]. In these studies, tasks were either multi-armed bandit problems [5,6,7,8,19] or an “observe and bet” task [11]. Nevertheless, as most environments change over time, to avoid misalignment with the contextual requirements and keep the energy cost optimally low, an adaptive strategy is required to balance between exploring and exploiting [20], and additional brain structures may be involved in this balance. For instance, according to the adaptive gain theory [21], the locus coeruleus not only drives norepinephrine release in the orbitofrontal cortex—which would permit estimating the reward—but also in the anterior cingulate cortex (ACC)—which would permit estimating the cost of these behaviors. The ACC is implicated in the evaluation of feedback and would promote either exploration in the case of negative feedback or exploitation if the feedback is positive [6]. Additionally, it is well-established that the cerebellum is engaged in motor learning processes, and there is increasing evidence that this is the case for cognitive tasks as well [22], notably in the building of internal models of stimuli–response associations [23]. The cerebellum, via cortico–cerebellar circuits, may also participate in the exploration/exploitation trade-off. Furthermore, it has been proposed that complex cognitive processes, such as behavioral adaptation or learning, rely on a continuous evolution of functional connections [24]. Although several theories propose that exploration and exploitation would be sustained by interactions among different cerebral areas, most studies have examined only isolated regions.

Functional connectivity (FC) analysis is a method that investigates functional communication between cerebral regions by measuring the synchronization of blood oxygen level-dependent (BOLD) signals, which are either task-related or spontaneous. In the latter case, intrinsic FC can be measured in participants who are in a mind-wandering state, defined as a “resting state” (Rs) [25]. Thus, this allows for the establishment of individuals’ intrinsic (i.e., basal, while resting) functional architecture [26].

Regarding task-related FC, we identified only one study that used a multi-armed bandit problem in non-treatment seeking gamblers and healthy controls in a set of regions of interest (frontal pole (FP), intraparietal sulcus, anterior insula, and dorsal ACC) [19]. Though the connectivity analyses confirmed that the pattern of functional network interactions could differentiate the group status, no significant group differences were found in the connectivity.

To our knowledge, only one study investigated the links between intrinsic/resting functional connectivity (rFC) and the exploration/exploitation trade-off [9]. Correlations between explorative behaviors and frontopolar cortex (FPC) connectivity were observed: the propensity to explore in the context of a reward was positively correlated with the connectivity between the FPC and the ventral striatum, while in the context of a loss, it was correlated with the connectivity between the FPC and precuneus. However, the rFC analyses focused on connections between a single region of interest (the FPC), and the whole brain, thus potentially overlooking correlations of connections excluding the frontopolar cortex. Moreover, whether the Rs acquisition was performed before or after the task is not specified in this study.

In line with the proposition that complex cognitive processes, such as behavioral adaptation or learning, rely on a continuous evolution of functional connections [24], an increasing number of studies have demonstrated that intrinsic functional architecture can be modified by coactivation of the brain’s areas even in a short-term period from before to after performing a task. These changes in resting functional connectivity (rFC) may inform on one’s short-term plasticity at the brain level, in other words, their basal neural flexibility [24]. For instance, learning has been linked to changes in rFC from before to after performing reinforcement learning problems in specific networks, a modulation that has been interpreted as a trace of memory consolidation [22,23,24,27,28,29,30,31]. In regard to our previous studies, we were also able to show that changes in rFC from before to after learning the correct associations between geometrical forms and a button response were linked to biological factors, such as age [28], but also to psychological factors, such as anxiety proneness or metacognitive skills [23]. It thus appears that such a methodology could be of great interest for capturing the modulation of functional connectivity linked to the adaptation to environmental uncertainty and its potential variability among individuals [28].

In sum, we found no study that investigated task-induced changes in rFC, nor any examining potential associations with interindividual factors, suggesting that much is still to be known regarding the neural correlates of the exploration/exploitation trade-off implementation. To fill in the gaps in the literature, the aims of the present study were twofold. The first goal was to characterize changes in rFC linked to behaviors of exploration or exploitation induced by unexpected changes in the environment. To do so, we used Rs fMRI resting-state acquisitions before and after a revised version of our associative learning task, which included a manipulation of the environmental uncertainty. As argued above, we expected to identify changes in rFC of the frontopolar cortex, ventromedial prefrontal cortex, orbitofrontal cortex, intraparietal sulcus, anterior cingulate cortex, insula, locus coeruleus, and the cerebellum. Our second aim was to explore psychological factors that are linked to behavioral flexibility, and which may thus relate to interindividual variability in balancing explorative and exploitative behaviors and the potential associated changes in rFC.

## 2. Materials and Methods

### 2.1. Participants

This study included volunteers recruited through community announcements. The inclusion and exclusion criteria were: an age between 18 and 60 years, right-handed, normal or corrected vision, normal IQ (as assessed by The French National Adult Reading Scale (f-NART [32,33]), no history of psychiatric or neurological conditions, and no MRI contraindication. The participants provided their written informed consent; they did not receive any monetary or material compensation for their participation.

**Figure 1:**
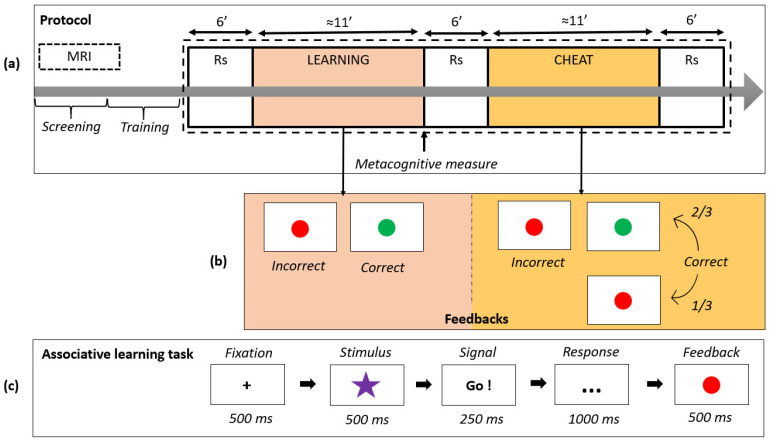
(**a**) Representation of the overall protocol: the resting state (Rs) scans before and after the CHEAT phases were used in the analyses. (**b**) Representation of the variations in feedbacks in the LEARNING and CHEAT phases. (**c**) Representation of the structure of a trial.

The overall procedure is described in Figure 1. On the same day, the participants first underwent a neuropsychological evaluation and then filled-out self-reported questionnaires. They further received a short training session to become familiar with the task before going into the MRI scanner.

### 2.2. Neuropsychological Tests and Psychometric Scales

We constituted a test battery of psychological dimensions related to behavioral flexibility and the monitoring of self-achievement. Accordingly, we chose to include a performance-based test of cognitive flexibility and self-report questionnaires of constructs related to the fear of failure and making mistakes, self-worth, and negative affect.

The participants’ cognitive flexibility was assessed using the Trail Making Test (TMT [34]), and the variable of interest was the difference in completion time between the form B and A; higher values are considered to reflect lower cognitive flexibility. For personality traits, the following questionnaires were used: the Frost Multidimensional Perfectionism Scale (FMPS [35]), the Social Self-Esteem Inventory (SSEI [36]), the Sensitivity to Punishment and Sensitivity to Reward Questionnaire (SPSRQ [37]), the State-Trait Anxiety Inventory (STAI-Y A et B [38]), and the Hospital Anxiety and Depression scale (HAD [39]). The subscales used are specified in Table 1. 

### 2.3. Associative Learning Task and MRI Protocol

The paradigm that we used was composed of two phases (Figure 1a): first, a classical associative learning phase (LEARNING) using a task that was drawn from Balsters and Ramnani [40], and a second phase during which the environmental uncertainty was introduced without informing the participants (CHEAT). Each phase had a duration of approximately 11 min, and the trials of each phase were organized in the same way (Figure 1b). Functional acquisitions were acquired during both phases of the task and interleaved with a 6 min Rs acquisition before and after each phase of the task. The overall task was computerized with E-prime software (v3.0; Psychological Software Tools, Sharpsburg, MD, USA).

During LEARNING, the participants learned to associate eight different geometrical forms to a single button among three. To do so, they proceeded by trial and error using the feedback given for each trial (Figure 1c): green for a correct combination and red for an incorrect one. This phase of the task terminated either when the participants achieved 80% accuracy in the sum of all trials or after a maximum of 300 trials. Once LEARNING ended, a metacognitive factor was measured by asking the participants to evaluate their level of confidence in their learning performance on a graduated scale (from 0 to 100%). Then, the participants were instructed to rest with their eyes closed but not to fall asleep (Rs acquisition). To introduce environmental uncertainty and trigger a balance between exploration and exploitation behaviors, false feedbacks were introduced in the second phase (CHEAT) of the paradigm. However, before the beginning of CHEAT, the participants were told they will be continuing the same task as before the resting period and were not informed that for one out of three correct answers, incorrect feedback would be given randomly (Figure 1c), suggesting to the participants that they gave an incorrect geometrical shape-response button association. In reality, the learned associations remained unchanged, and feedback signaling an incorrect answer was given randomly in 1 out of 3 trials despite a correct answer. For all the participants, the CHEAT phase terminated after 240 trials.

### 2.4. MRI Acquisition

#### 2.4.1. Acquisition

Anatomical and functional brain imaging data were acquired using a Siemens Prisma MRI. Anatomical images were obtained with a 3D MPRAGE T1-weighted MRI sequence with the following parameters: repetition time (TR) = 2000 ms, echo time (TE) = 2 ms, field of view (FOV) = 256 × 256 mm^2^ to cover the entire brain, 192 slices, and an isotropic voxel size = 1 × 1 × 1 mm^3^. Resting functional images were acquired with a multiband sequence according to the following parameters: TR = 1000 ms, TE = 30ms, FOV = 220 × 220 mm^2^, 60 slices, 360 dynamics, voxel size = 2.5 mm^3^. All acquisitions were realigned along the anterior commissure–posterior commissure (AC-PC) plane.

#### 2.4.2. Preprocessing

The anatomical scans and functional images were preprocessed using SPM12 (https://www.fil.ion.ucl.ac.uk/spm/software/spm12/, accessed on 20 April 2022) and the CONN toolbox (https://web.conn-toolbox.org/, accessed on 20 April 2022). For each participant, the functional images were first realigned to the middle scan to correct for small movements that might have occurred during the acquisition. A mean functional image was created at this step, and the anatomical image was coregistered on this mean and segmented, enabling the later use of an anatomical grey matter image for the normalization of the functional images. Then, the functional images were warped in the same space, the MNI template (MNI 152, Montreal Neurological Institute, McGill University, Montreal, Canada). The grey matter volume was used to define the normalization parameters. The normalization parameters were applied to the functional images, which were then smoothed using a FWHM of 5mm to correct for BOLD signal artifacts at the voxel level. The functional images were then detrended to correct for the loss of signal along the acquisition, and signals from the white matter and cerebrospinal fluid regions were extracted using principal component analysis. Then, the functional volumes were band-pass filtered (between 0.009 and 0.08 Hz) to ensure that the analyses were completed within the frequency band of interest. Finally, the functional images were despiked to remove outliers in the time-series signal.

### 2.5. Statistical Analyses

#### 2.5.1. Behavioral Data

All statistical analyses were carried out using R Statistical Software (v4.1.3; R Core Team, 2022) and jamovi software (v2.3.21, The jamovi project, 2022).

To first study the overall participants’ behaviors along the two phases of the task, we defined four periods of interest in the evolution of accuracy. These periods of interest were the first and last quartiles of the trials of both the LEARNING and CHEAT phases. For the LEARNING phase, as the number of trials varied as a function of the level of accuracy (mean = 218 ± 78.8, min–max = 90–300), the number of trials used to define the two periods of interest of this phase differed among the participants (mean = 46 ± 18.5, min–max = 17–74). For the CHEAT phase, as the number of trials was fixed for all the participants (240 trials), the first and last 60 trials were used to define the two periods of interest of this phase.

As a prerequisite, we analyzed how the accuracy evolved across the different phases of the task. The variables of interest were the mean accuracies (%) during each of the four periods of interest (first and last quartiles of the task) and were analyzed using a contrast analysis. The principal contrast was quadratic with a cubic tendency, and the two residuals’ contrasts were linear.

Then, for each participant, an *exploitation index* was inferred using the accuracy during the CHEAT phase by calculating the mean accuracy only in the trials involving the stimuli (geometrical figures) for which the participants’ accuracy reached at least 80% of correct responses at the end of LEARNING (last quartile of the LEARNING trials). The accuracy in a partially uncertain environment reflected how the participants exploited known associations. A low *exploitation index* reflects that the participants who were less accurate in regard to previously learned combinations explored other associations.

Moreover, regarding the reaction to unexpected uncertainty, the difference between the mean accuracy at the beginning of CHEAT (first quartile of the trials) and that at the end of LEARNING (last quartile of the trials) was calculated. This Δ was defined as the *cost of uncertainty*, and higher values reflect more explorative behaviors in reaction to the introduction of cheated feedbacks. Two groups of participants were created using this latter variable and a median split categorization: “Cost+” and “Cost−” groups. Between-group comparisons were realized to assure that the groups were matched for both demographical and psychological variables (Table 1). Qualitative variables were compared to chi-squared tests (effect size described with Cramer’s V). Continuous variables were compared with Student’s *t*-tests (effect size described with Cohen’s d) when conditions of normality were met; otherwise, Mann–Whitney’s U tests were performed (effect size described with rank biserial correlations).

#### 2.5.2. fMRI Data

**Figure 2:**
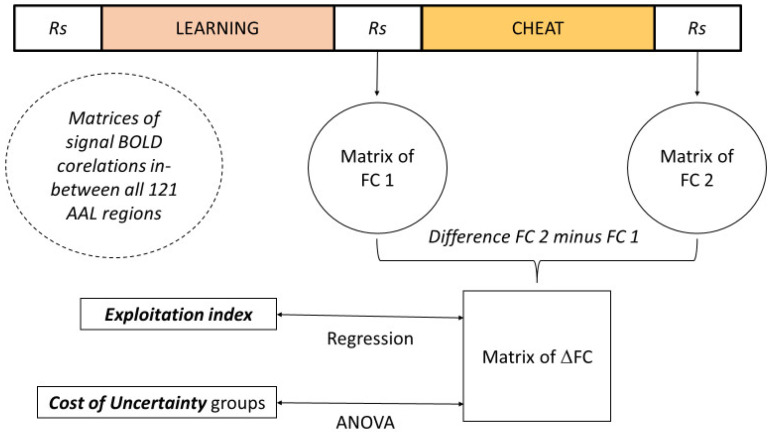
Description of fMRI analyses of interest.

All analyses of the connectivity data were run using the CONN toolbox (version 16, MIT, Cambridge, MA, USA) [41] and described in Figure 2. The mean BOLD signal was first measured in a parcellated brain of 116 regions (automated anatomical labelling, [42]). Then, a functional connectivity (FC) matrix of correlations among the BOLD signals extracted in the AAL regions was constructed for the resting-state (Rs) acquisitions (before and after the CHEAT phase).

Whole-brain changes in the Rs functional connectivity values (RsFC) induced by the CHEAT phase were measured by subtracting the RsFC values of the post-CHEAT period from those of the pre-CHEAT period, resulting in a Δ value (ΔFC). It was used as a within-subject variable in the two following analyses run in the CONN toolbox. First, a contrast between the “Cost+” and “Cost−” groups was carried out. This analysis was equivalent to an ANOVA, resulting in pairs of regions for which the FC differed between the two groups. Then, the level of *exploitation index* was defined as a between-subject variable. This analysis was equivalent to a regression, with the *exploitation index* as the predictor variable and the ΔFC as the outcome variable.

All results were corrected for multiple comparison using a false discovery rate (FDR) correction with a α level of 0.05.

#### 2.5.3. Interindividual Variability

Associations among the neuropsychological tests or psychometric scales scores and the individual markers of the exploration/exploitation trade-off at the behavioral level (*cost of uncertainty index*) and at the cerebral level (Δ*FC*) were tested using Spearman correlations. To correct for multiple comparisons, a Bonferroni–Holm correction was applied.

## 3. Results

Thirty-six participants were recruited for the protocol. Three were considered outliers and excluded from behavioral analysis due to a low level of accuracy during LEARNING (i.e., mean accuracy inferior to that of the overall sample minus two standard deviations). Two participants were excluded because of high movements during the Rs acquisitions, and three others did not stay in the scanner for the last Rs acquisition. Data from the remaining 28 participants (15 women, 13 men) were included for the behavioral and connectivity analyses. Most of the participants (74%) had a level of education equal or superior to three years after the French high school diploma (i.e., Baccalauréat), 15% had a level of education of two years after their Baccalauréat degree, and 11% only had their Baccalauréat degree. For the analyses investigating the links with the psychological variables, the number of participants varied due to incomplete data (Table 1).

### 3.1. Descriptive of Psychological Variable

Descriptive statistics of the psychological variables for the whole sample and by subgroup (“Cost+” and “Cost−”) are presented in Table 1. The “Cost+” (n = 14; 6 men) and “Cost−“ (n = 14; 7 men) groups did not differ regarding sex repartition, age, education level (for the “Cost+”/“Cost−” groups, respectively, 79/69% had three or more years of education after their Baccalauréat degree, 7/23% had a level of education of two years after their Baccalauréat degree, and 14/8% only had their Baccalauréat degree), or for any of the psychological variables of interest.

### 3.2. Behavioral Analyses

#### 3.2.1. Overall Accuracy

**Figure 3:**
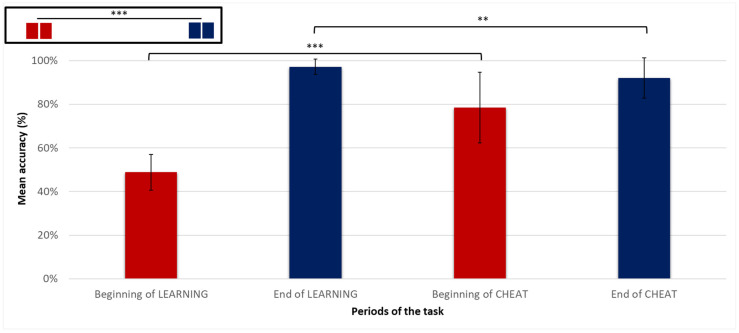
Mean accuracy (%) in the four periods of interest for all participants (***: *p* < 0.001; **: *p* < 0.05).

For LEARNING, the mean accuracy evolved from 48 ± 9% at the beginning to 97 ± 3% at the end of this phase. For CHEAT, it evolved from 78 ± 16% at the beginning to 92 ± 9% at the end of this phase (Figure 3). Regarding the contrast analyses between the means of accuracy at the four periods of interest in the two phases of the task, the principal contrast showed that the mean accuracy at the beginning of both LEARNING and CHEAT was significantly inferior to the mean accuracy at the end of these phases (T(31) = 20.21; *p* < 0.001). The residuals’ contrasts showed that the mean accuracy at the beginning of LEARNING was significantly lower than the mean accuracy at the beginning of CHEAT (T(31) = 8.39; *p* < 0.001), and that the mean accuracy at the end of CHEAT was significantly lower than the mean accuracy at the end of LEARNING (T(31) = −2.98; *p* = 0.01). These results reflect the expected effect of uncertainty’s introduction and allowed us to further study the exploration/exploitation trade-off. 

**Figure 4:**
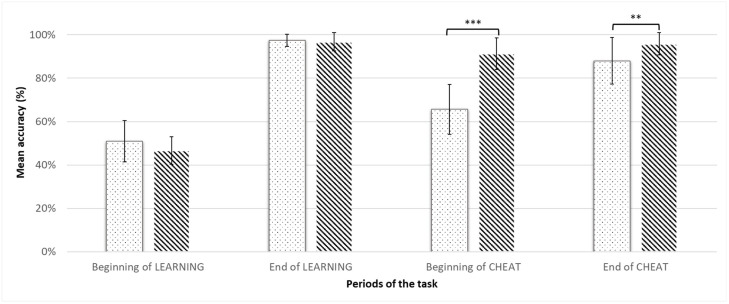
Mean accuracy (%) in the four periods of interest by subgroups (Mean accuracy of “Cost+” group in dotted bars and mean accuracy of “Cost−” group in striped bars.) (***: *p* < 0.001; **: *p* < 0.05).

We performed a mixed ANOVA to study the interaction between the groups (“Cost+” and “Cost−”) and the periods of the task (Figure 4). There was a significant main effect of the group only at the beginning of CHEAT (F(26) = 49.4, *p* < 0.001) and at the end of CHEAT (F(26) = 5.99, *p* = 0.02), with the accuracy being inferior in the “Cost+” group.

#### 3.2.2. Cost of Uncertainty and Exploitation Indices

Between the end of LEARNING and the beginning of CHEAT, the *cost of uncertainty* represented a decrease in accuracy equal to 19 ± 16%. The participants were separated into two groups using a median split of this variable: participants with a high *cost of uncertainty* were in the “Cost+” and those with a low *cost of uncertainty* were in the “Cost−” group (Figure 4). Thus, the “Cost+” group had a mean decrease in accuracy of 32 ± 12%, while the “Cost−” group had a mean decrease in accuracy of 5 ± 6%. The overall mean of the *exploitation index* was equal to 87 ± 11%, and was equal to 94 ± 0.05% for the “Cost−” group and 79 ± 0.1% for the “Cost+” group.

### 3.3. Resting-State Functional Connectivity

**Figure 5:**
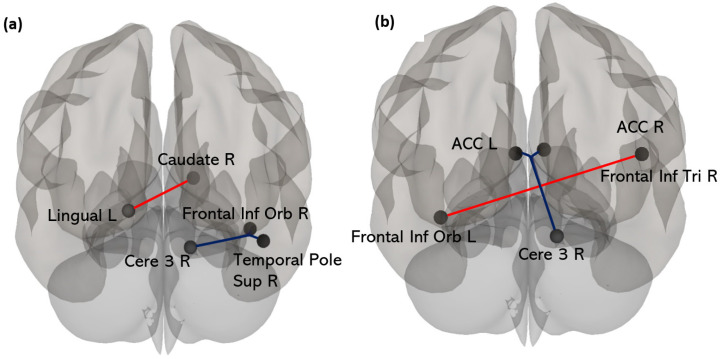
Posterior view representations of the connectivity analyses. (**a**) Group differences in changes in functional connectivity (ΔFC): FC values increasing in the “Cost+” group and decreasing in the “Cost−” are displayed in red; FC values increasing in the “Cost−” group and decreasing in the “Cost+” are displayed in blue. (**b**) Changes in functional connectivity significantly associated with the *exploitation index:* positive correlations are displayed in red and negative correlations in blue. R: right; L: left; ACC: anterior cingulate; Frontal Inf Tri: triangular part of the frontal inferior gyrus; Temporal Pole Sup: superior pole of the temporal lobe; Frontal Inf Orb: orbital part of the frontal inferior gyrus; Cere 3: cerebellar region 3; Caudate: caudate nuclei; Lingual: lingual gyri.

The ANOVA between the “Cost+” and “Cost−” groups revealed significant differences in the ΔFC in three pairs of regions (Figure 5a). On average, the changes in connectivity were in opposite directions between the “Cost+” and “Cost−” groups. These included:

(i). The ΔFC between the right caudate and left lingual gyrus (T(26) = 4.52, *p* < 0.001, *p*_(FDRCorr)_ = 0.01, d = 1.17), where connectivity increased in the “Cost+” (ΔFC = 0.09 ± 0.17) but decreased in the “Cost−” (ΔFC = −0.12 ± 0.15) group;
(ii). The ΔFC between the right cerebellar region 3 and the right superior pole of the temporal lobe (T(26) = −4.49, *p* < 0.001, *p*_(FDRCorr)_ = 0.01, d = 1.70), where connectivity decreased in the “Cost+” (ΔFC = −0.10 ± 0.15) group but increased in the “Cost−” (ΔFC = 0.10 ± 0.13) group;
(iii). The ΔFC between the right cerebellar region 3 and the orbital part of the right frontal inferior gyrus (T(26) = −3.78, *p* < 0.001, *p*_(FDRCorr)_ = 0.05, d = 1.43), where connectivity decreased in the “Cost+” (ΔFC = −0.11 ± 0.07) group, but increased in the “Cost−” (ΔFC = 0.08 ± 0.21) group.

In the overall sample, the exploitation index and ΔFC between the right cerebellar region 3 and both the right and left anterior cingulate cortices (ACC) were negatively associated (respectively, T(29) = −3.90, *p*_(FDRCorr)_ = 0.04; T(29) = −3.82, *p*_(FDRCorr)_ = 0.04). The change in connectivity between these areas (ΔFC = −0.13 ± 0.19) corresponded to an increase in an anticorrelation (present before CHEAT), such that the more the participants explored, the more anticorrelated the connection was between the two areas. Moreover, the exploitation index and ΔFC between the orbital part of the left frontal inferior gyrus and the triangular part of the right frontal inferior gyrus were positively associated (T(29) = 3.99, *p*_(FDRCorr)_ = 0.054). The change in connectivity between these areas corresponded to an increase in their positive correlation (ΔFC = 0.13 ± 0.22) (Figure 5b), such that the more the participants exploited, the stronger the connections were. However, non-significant but non-negligible negative associations were found between the TMT B-A and ΔFC between the right cerebellar region 3 and the right superior pole of the temporal lobe (r = −0.34) and between the right cerebellar region 3 and the orbital part of the right frontal inferior gyrus (r = −0.37).

### 3.4. Associations with the Psychological Variables

No significant correlation was found between the *exploitation index* or the *cost of uncertainty* variable and any of the psychological variables investigated (See Table S1). However, both indices showed correlations of non-negligible rho values (i.e., above 0.2), with some variables, notably the TMT B-A, the STAI Trait, and the HAD Depression and Anxiety scores.

Regarding the changes in connectivity, in the overall sample, the ΔFC between the right orbital part and the left triangular part of the inferior frontal gyrus was significantly positively correlated with the “*Doubts about actions*” (r = 0.44, *p* = 0.03) and the HAD *Anxiety* (r = 0.45, *p* = 0.03) scores. Moreover, the HAD *Anxiety* score was positively correlated with the ΔFC between the cerebellum and both the right (r = 0.48, *p* = 0.02) and left (r = 0.53, *p* = 0.01) ACC. Yet, these associations did not remain significant after the Bonferroni–Holm adjustment. Additional non-significant but non-negligible associations (rho values ranging from +/− 0.27 to 0.36) were found.

Concerning the three pairs of regions for which the ΔFC were found to differ between the “Cost+” and the “Cost−” groups, none were found significantly correlated with any of the psychological variables (See Table S2). However, non-significant but non-negligible negative associations were found between the TMT B-A and ΔFC between the right cerebellar region 3 and the right superior pole of the temporal lobe (r = −0.34) and between the right cerebellar region 3 and the orbital part of the right frontal inferior gyrus (r = −0.37).

## 4. Discussion

In the present study, we identified changes in intrinsic cerebral connectivity from before to after performing a task that induced unexpected uncertainty, and we were able to link these changes to inter-individual variability in balancing explorative and exploitative behaviors, anxiety, and perfectionism proneness.

Overall, at the behavioral level, the introduction of unexpected uncertainty using false feedbacks induced explorative behaviors. This being said, the proportion of explorative behaviors was modest. This finding is in agreement with Walker et al. [43], who showed that if the participants had an opportunity to learn the structure of the task before experiencing uncertainty, they would not explore as much as expected when the environment changed. Here, during CHEAT, false feedbacks occurred in only one-third of the trials. Hence, statistically speaking, the environment was globally stable, with a context where the majority of the expected positive feedbacks occurred, a context that may challenge the potential gain of exploration (versus exploitation) to remain efficient. Future studies should help to determine if an increase in the number of false feedbacks significantly impacts the exploration/exploitation trade-off.

At the brain level, there are some differences among the regions for which we hypothesized some changes in rFC based on the literature and those identified in our analyses. We did not find rFC changes in all the frontal subregions reported in the literature. Yet, in these studies, the connectivity levels were measured either during the task or during rest but at a distance from the task. In the present case, changes in connectivity were explored from before to after tasking, and interestingly, they were significant between the frontal subregions, which are very close to the ones reported previously.

Our results do not overlap either with the intraparietal sulcus, the insula, or the locus coeruleus. Here, we speculate that the difference in the type of tasks used may explain why our hypotheses were not fully validated. Indeed, in the present study, a trial–error learning paradigm was used, whereas in most of the previous ones, a high reward-based learning (gain/loose) paradigm was adopted. Regarding the locus coeruleus specifically, its small size may also have prevented us from observing changes in rFC. Yet, the present findings highlight regions coherently associated with the exploration/exploitation trade-off.

Expanding on previous studies that showed that the basal cerebral state, as indexed by intrinsic FC, is modified in the short term after the brain has been engaged in consolidating learned action–outcome contingencies [23,24,27,29,30,31,44], the present study showed that it also occurs after being engaged in the remapping of previously learned action–outcome contingencies. Moreover, as we speculated previously [23], modifications in intrinsic regional connectivity differed depending on the behavioral tendencies adopted by the participants when facing unexpected changes in the environment.

The participants with the highest performance cost (i.e., those who adapted with a greater number of explorative behaviors) due to the introduction of false feedbacks (“Cost+” group) had an increase in intrinsic connectivity between the right caudate nucleus and the left lingual gyri, while the connectivity between these two regions decreased among the participants with the lowest performance cost (“Cost−” group). The caudate nucleus, which is part of the striatum and basal ganglia, is involved in the dopaminergic system. Dopamine has an influence on the implementation of explorative behaviors, mediated by the neural coding of uncertainty [7]. Regarding the lingual gyrus, studies have shown that it is involved in visual processing and that it may be more particularly involved in visual memory [45,46]. Furthermore, Callan et al. [47] found that the lingual gyrus is involved in tracking the resolution of uncertainty, and Bruguier et al. [48] reported enhanced lingual gyrus activation when trading risk increased in a financial market’s context. This region might therefore be involved in uncertainty processing per se.

In parallel with the above-mentioned network, changes in cerebellar connectivity were also linked to the degree individuals balanced between explorative and exploitative behaviors when their learned action–outcome contingencies were challenged. The literature suggests that cortico–cerebellar loops could be involved not only in learning but also in the adaptation of learning processes [49]. These loops convey feedforward information from the cortex to reach the cerebellum, but also reverse feedback information to circulate [50] and enable the formation of cerebellar internal models [51]. Internal models would represent the context associated with a motor action used by the organism to predict and anticipate sensory-motor feedback from the environment. This conceptualization can also be applied to non-motor learning: cognitive processes can also be modelized and associated to environmental or even mental stimuli [51]. This anticipation is required to generate rapid, automatic, and adaptive behaviors [49]. Our interpretation of the opposite pattern of associations between those who explore (“Cost+”) versus those who exploit (“Cost−”) is that the former group disengaged the cerebellum to re-map their internal models, while the latter re-engaged the cerebellum to rely upon the internal models built during learning the action–outcome contingencies (i.e., the first phase of the task).

This interpretation is supported by the regression analysis of the whole sample. Two pairs of regions were linked with the exploitation propensity and changes in functional connectivity from before to after the CHEAT phase of the task.

First, a low propensity to exploit was linked to an increase in anti-correlation between the ACC and the cerebellum connectivity. Due to the task dichotomization of behaviors (accurate or inaccurate answers), a low exploitation index implies a high exploration. Thus, a high rate of explorative behavior relates to high changes in FC, which, in this case, was an anticorrelation. The area of the cingulate cortex that we found here overlaps with the one described by Behrens et al. [52] as correlating with volatility (i.e., the unconditional probability of a jump), as well as with the estimation of uncertainty. In addition, the implication of the ACC in explorative behaviors has been brought up by studies showing higher activation in explorative > exploitative contrasts [5,7,11]. The activation of this area could thus reflect its involvement in the evaluation of uncertainty [6], promoting explorative behaviors when necessary. The ACC could be involved either in the detection or resolution of conflicts, and thus, when stereotyped behaviors need to be replaced by novel behaviors. More precisely, the anticorrelation of the FC between the ACC and the cerebellum has been interpreted as an inhibition of stereotyped responses [53]. In the context of the present study, we observed similar results, which may reflect the inhibition (from the ACC on the cerebellum) of internal models representing previously learned combinations that are supposedly set up in the cerebellum [51]. To explore new possibilities, the participants had to inhibit the use of combinations they had learned during the LEARNING phase of the task, and thus, adjust the corresponding internal models. Nevertheless, this interpretation needs to be considered carefully, as FC cannot inform about the directionality of connections. Moreover, other studies have reported activation in the cerebellum associated with explorative behaviors when contrasted with exploitative ones [7,8]. We must highlight that the exact location of such activation in the cerebellum is not specified. Considering the present study, cerebellar region 3 seems to play a critical role. Little information about this specific anterior part of the cerebellum was found in the literature, but it is mainly considered as being related to sensorimotor processing [50]. For instance, motor impairment is associated with lesions of the anterior lobes of the cerebellum, while cognitive or emotional impairments have been associated with lesions in posterior cerebellar regions [54]. Herein, internal models of learned combinations could relate to cerebellar motor areas since the task enables the automatization of this learning [40].

The regression analysis also revealed a positive association between a higher propensity to exploit and an increase in the connectivity between the right and the left frontal inferior gyri. These areas are associated with the inhibition of non-pertinent stimuli [55]. For subjects who adopted exploitation behaviors regardless of the introduction of false feedbacks, irrelevant information during the CHEAT phase had to eventually be ignored. Thus, such modulation of frontal FC could reflect how the participants incorporated the occurrence of false feedback in their existing models as an “expected uncertainty”. In doing so, the participants could exploit rather than explore. These results may reflect task-specific processes, but, in a more general way, exploitation may depend on an inhibition of environmental uncertainty.

Finally, associations were found between the psychological variables and the level of changes in functional connectivity statistically linked to the behavioral variable reflecting exploitation (i.e., the *exploitation index*).

First, changes in functional connectivity between the bilateral ACC and the right cerebellar region 3 were found to be significantly and positively linked with the level of anxiety (as measured by the HAD scale). These changes in FC were an increase in anti-correlation, meaning that greater levels of anxiety corresponded to lesser changes, in other words, to lesser basal neural plasticity. This suggestion is somewhat supported by the negative (though not significant) associations between these changes in rFC and the TMT B-A, where higher values reflect lower cognitive flexibility. On the other hand, the increase in the connectivity between the orbital part of the left frontal inferior gyrus and the triangular part of the right frontal inferior gyrus also correlated positively with the level of anxiety. Depending on the context, exploring could be linked with lower levels of anxiety since it allows one to gain more information about the environment [56], or, conversely, turning aside from routines and exploring uncertain alternatives could be associated with an increased experience of anxiety [57]. Although anxiety is often conceptualized as maladaptive, evolutionary theorists consider fear and anxiety as functional aspects of mammalian evolution [58]. Indeed, anxiety has been linked to increased exploitation of information for food acquisition in mice [59], and anxiety predicts low-risk, low-reward decision-making under risk in humans [60]. Thus, it may be that individuals who are prone to experience anxiety may fare best in contexts optimized for exploitation over exploration [61]. In this study, it seems that a high level of anxiety relates to higher changes in rFC between the frontal regions but with fewer changes between the cerebellum and cortex. Anxiety did not significantly correlate with the behavioral scores, but since non-negligible coefficients of correlation were found between the different measures of anxiety and the behavioral indices, future studies including larger samples are needed to clarify this issue. Nonetheless, it seems that anxiety could be associated with different patterns of network plasticity among the cerebral regions linked with the exploration/exploitation trade-off.

A frontofrontal increase in connectivity also correlated positively with the FMPS score “Doubts about actions” (DA), a dimension of perfectionism. The DA dimension defines how participants tend to hesitate in acting. The more precautious the participants were, the higher the FC changes were between the two frontal cortices. It seems that while perfectionism is not directly linked to a behavioral tendency to exploit, it is positively associated with neural plasticity among regions whose change in FC relates to exploitation.

From the present work, some perspectives but also limitations should be considered. A first potential limit of our study is that we did not use a classical measure of explorative and exploitative behaviors (e.g., multi-armed bandit problem [4]), which may restrain the comparison of our findings to other studies. However, the diversity of tasks used and their variations can already restrict comparisons among studies [12]. Herein, the use of an associative learning task allowed us to study the trade-off in a low-level cognitive task with rapidly automatized responses. Thus, the use of this task could permit the trigger of switches between exploration and exploitation behaviors even when the individual is not making an explicit choice. With this task, we found additional arguments for stating that the use of false feedback is relevant to introducing uncertainty in a learning environment.

Regarding our observations of interindividual differences in reactions to a manipulation that induced a rather low level of environmental uncertainty (as only one-third of the correct responses were cheated), replicating this study by increasing the level of uncertainty may permit researchers to further examine if interindividual variability increases with greater environmental volatility. Moreover, in regard to the inter-correlations among the behavioral, psychological, and neural variables, our results point to the probable influence of anxiety proneness and some dimensions of perfectionism, and maybe of cognitive flexibility (at least as indexed by the TMT B-A). Yet, given the rather small sample size and little variance in the scores of interest, these suggestions and the validity of the exploitation and uncertainty indices need to be confirmed on a larger population with more diversityin terms of these personality traits.

Altered implementation of the balance between explorative and exploitative behaviors may occur in many psychiatric pathologies, and impaired adaptation to one’s environment could reflect more ecological repercussions of a disorder [62]. Interestingly, pathologies, such as addiction or schizophrenia, are known to be linked to both impairment in the implementation of the exploration/exploitation trade-off [9,15] and cerebellar dysfunctions [49]. As internal models are sustained by cortico–cerebellar loops [51], cerebellar impairments could, therefore, restrain the use of these models in an adaptive way.

In addition, using such a paradigm does not permit drawing any conclusion on time-locked processes, and our findings may lack specificity because rFC changes could be linked to different phases of the task, such as feedback processing or association recalling. However, this study highlights interindividual differences in basal network plasticity in relation to environmental changes and personality traits. In regard to our findings on the changes in functional connectivity, different regions of interest linked to the exploration/exploitation trade-off could be explored in a more pin-pointed way. For instance, much is still to be learned about the functional implications of cerebellar region 3.

## 5. Conclusions

This study examined changes in brain connectivity and behavioral responses to unexpected uncertainty. We found that the introduction of uncertainty led to exploratory behaviors, although the proportion of such behaviors was modest. Changes in intrinsic brain connectivity differed according to individual behavioral tendencies. Participants with more exploratory behaviors showed increased connectivity between the right caudate nucleus and the left lingual gyrus, while participants with fewer exploratory behaviors showed reduced connectivity between these regions. In addition, changes in cerebellar network connectivity were associated with how individuals balanced exploratory and exploitative behaviors in response to uncertainty in their learning environment.

The study also examined the relationship between psychological variables and changes in functional connectivity. The results indicate that anxiety levels tend to be positively correlated with changes in connectivity between the anterior cingulate cortex (ACC) and the cerebellum. In addition, increased connectivity between the orbital part of the left inferior frontal gyrus and the triangular part of the right inferior frontal gyrus also tended to correlate with anxiety scores. Perfectionism, as measured by the FMPS, also tended to be positively correlated with increased connectivity between these frontal cortices. These correlations highlight the inter-individual variability of network plasticity related to the exploration/exploitation trade-off and its associations with anxiety and perfectionism.

## Figures and Tables

**Table 1:** Descriptive statistics of the psychological variables.

	ALL		COST−		COST+		Group Comparison
	Mean (sd)	n	Mean (sd)	n	Mean (sd)	n	Statistics *
Age (years)	30.00 (10.87)	28	32.21 (13.47)	14	27.79 (7.32)	14	U = 91.00; *p* = 0.76; r = 0.41
TMT B-A (seconds)	21.69 (12.93)	24	18.28 (10.26)	14	26.47 (15.22)	10	U = 43.00; *p* = 0.12; r = 0.39
FMPS_CM	23.00 (8.59)	25	23.23 (9.76)	13	22.75 (7.55)	12	t = 0.14; *p* = 0.89; d = 0.05
FMPS_DA	11.40 (4.00)	25	11.62 (3.57)	13	11.17 (4.58)	12	t = 0.28; *p* = 0.79; d = 0.11
FMPS_O	22.24 (5.77)	25	22.92 (5.02)	13	21.50 (6.63)	12	t = 0.61; *p* = 0.55; d = 0.24
FMPS_PS	22.20 (5.42)	25	21.69 (4.80)	13	22.75 (6.18)	12	t = −0.48; *p* = 0.64; d = 0.13
SPSRQ_P	43.64 (10.05)	28	42.36 (9.71)	14	44.93 (10.58)	14	t = −0.67; *p* = 0.51; d = 0.11
SPSRQ_R	37.61 (6.05)	28	37.00 (7.32)	14	38.21 (4.64)	14	t = −0.52; *p* = 0.60; d = -0.20
SSEI	129.92 (21.67)	25	131.29 (18.92)	14	128.18 (25.60)	11	U = 76.00; *p* = 0.98; r = 0.01
STAI_S	31.89 (9.69)	28	30.71 (8.51)	14	33.07 (10.94)	14	U = 87.00; *p* = 0.63; r = 0.11
STAI_T	42.10 (12.88)	28	43.00 (13.93)	14	41.14 (12.19)	14	t = 0.36; *p* = 0.71; d = 0.14
HAD_D	4.91 (3.79)	23	6.00 (4.59)	12	3.73 (2.323)	11	U = 52.50; *p* = 0.42; r = 0.20
HAD_A	6.57 (4.25)	23	6.33 (3.37)	12	6.82 (5.21)	11	U = 56.50; *p* = 0.57; r = 0.14
Confidence in learning (%)	77.37 (6.05)	28	76.55 (6.76)	14	78.18 (5.36)	14	U = 83.00; *p* = 0.50; r = 0.15

* Student’s *t*-test was performed when conditions were applicable, otherwise Mann–Whitney U test was performed. TMT: Trail Making Test; FMPS: Frost Multidimensional Perfectionism Scale (CM: “Concern over Mistakes”, DA: “Doubts about Actions”, O: “Organization”, PS: “Personal Standards”); SPSRQ: Sensitivity to Punishment and Sensitivity to Reward Questionnaire (P: “Sensitivity to Punishment”, R: “Sensitivity to Reward”); SSEI: Social Self-Esteem Inventory; STAI: State-Trait Anxiety Inventory (S: “State”, T: “Trait”); HAD: Hospital Anxiety and Depression scale (A: “Anxiety” and D: “Depression”).

## Data Availability

All data are available upon request.

## Supplementary Materials

The following supporting information can be downloaded at: https://www.mdpi.com/article/10.3390/biology12101323/s1, Table S1: Correlation matrix of behavioral and psychological variables; Table S2: Correlation matrix of changes of rFC and psychological variables.
