# Supplementary material for: Interindividual Brain and Behavior Differences in Adaptation to Unexpected Uncertainty
Source: Biology (Basel). 2023 Oct 10;12(10):1323. doi: 10.3390/biology12101323 (PMC10604029; doi:10.3390/biology12101323)
Supplement: Supplementary file 1 [file biology-12-01323-s001.zip › biology-2493797-supplementary.pdf]

Table S1. Correlation matrix of behavioral and psychological variables

		Exploi	Cost	Confidence	TMT B-A	FMPS_PE	FMPS_DA	FMPS_O	FMPS_SP	SPSRQ_P	SPSRQ_R	STAI_E	STAI_T	SSEI	HAD_D	HAD_A
Exploi	Spearman's rho	—														
	valeur p	—														
Cost	Spearman's rho	-0.863 ***	—													
	valeur p	< .001	—													
Confidence	Spearman's rho	-0.160	0.217	—												
	valeur p	0.415	0.267	—												
TMT B-A	Spearman's rho	-0.224	0.252	-0.207	—											
	valeur p	0.292	0.234	0.332	—											
FMPS_PE	Spearman's rho	0.128	-0.101	0.030	-0.191	—										
	valeur p	0.543	0.631	0.888	0.382	—										
FMPS_DA	Spearman's rho	0.125	-0.124	-0.029	0.061	0.562 **	—									
	valeur p	0.551	0.555	0.889	0.784	0.003	—									
FMPS_O	Spearman's rho	0.127	-0.150	-0.305	0.285	-0.225	0.195	—								
	valeur p	0.547	0.474	0.139	0.188	0.280	0.349	—								
FMPS_SP	Spearman's rho	-0.047	0.112	0.228	-0.259	0.607 **	0.473 *	-0.035	—							
	valeur p	0.822	0.593	0.274	0.232	0.001	0.017	0.869	—							
SPSRQ_P	Spearman's rho	0.248	-0.117	0.026	-0.057	0.535 **	0.596 **	0.177	0.353	—						
	valeur p	0.202	0.552	0.894	0.791	0.006	0.002	0.397	0.083	—						
SPSRQ_R	Spearman's rho	-0.156	0.110	0.591 ***	-0.108	0.268	0.229	-0.119	0.327	-0.014	—					
	valeur p	0.429	0.576	<.001	0.616	0.196	0.271	0.572	0.111	0.945	—					
STAI_E	Spearman's rho	0.284	-0.116	0.041	0.210	0.335	0.193	-0.075	0.108	0.648 ***	-0.136	—				
	valeur p	0.143	0.556	0.835	0.326	0.101	0.355	0.722	0.608	< .001	0.490	—				
STAI_T	Spearman's rho	0.293	-0.204	0.051	-0.129	0.742 ***	0.496 *	-0.093	0.460 *	0.765 ***	0.060	0.656 ***	—			
	valeur p	0.131	0.298	0.797	0.549	< .001	0.012	0.659	0.021	< .001	0.761	< .001	—			
SSEI	Spearman's rho	-0.276	0.132	-0.131	-0.023	-0.364	-0.637 **	-0.215	-0.170	-0.835 ***	0.010	-0.561 **	-0.684 ***	—		
	valeur p	0.182	0.529	0.533	0.918	0.087	0.001	0.325	0.438	< .001	0.961	0.004	< .001	—		
HAD_D	Spearman's rho	0.265	-0.220	0.065	0.139	0.468 *	0.334	0.064	0.326	0.412	-0.020	0.359	0.487 *	-0.302	—	
	valeur p	0.222	0.313	0.767	0.547	0.032	0.140	0.782	0.149	0.051	0.927	0.093	0.019	0.173	—	
HAD_A	Spearman's rho	0.210	-0.237	-0.355	-0.132	0.567 **	0.368	0.124	0.188	0.589 **	-0.102	0.460 *	0.685 ***	-0.439 *	0.304	—
	valeur p	0.336	0.277	0.097	0.569	0.007	0.101	0.593	0.415	0.003	0.642	0.027	< .001	0.041	0.158	—

*Legend : * p < .05, ** p < .01, *** p < .001

Confidence : confidence in learning (%); **TMT:** Trail Making Test; **FMPS**: Frost Multidimensional Perfectionism Scale (CM: “Concern over Mistakes”, DA: “Doubts about Actions”, O: “Organization”, PS: “Personal Standards”); **SPSRQ**: Sensitivity to Punishment and Sensitivity to Reward Questionnaire (P: “Sensitivity to Punishment”, R: “Sensitivity to Reward”); **SSEI**: Social Self-Esteem Inventory; STAI: State-Trait Anxiety Inventory (S: “State”, T: “Trait”); **HAD**: Hospital Anxiety and Depression scale (A: “Anxiety” and D: “Depres-sion”).

Table S2. Correlation matrix of changes of rFC and psychological variables

		front_front	cere_cinguR	cere_cinguL	cere_tempo	cere_front	cauda_lingu	Confidence	TMT B-A	FMPS_PE	FMPS_DA	FMPS_O	FMPS_SP	SPSRQ_P	SPSRQ_R	STAI_E	STAI_T	SSEI	HAD_D	HAD_A
front_front	Spearman's rho	—																		
	p-value	—																		
cere_cinguR	Spearman's rho	0.108	—																	
	p-value	0.585	—																	
cere_cinguL	Spearman's rho	0.236	0.755 ***	—																
	p-value	0.226	< .001	—																
cere_tempo	Spearman's rho	0.327	0.326	0.384 *	—															
	p-value	0.090	0.090	0.044	—															
cere_front	Spearman's rho	0.278	0.180	0.294	0.641 ***	—														
	p-value	0.152	0.359	0.129	< .001	—														
cauda_lingu	Spearman's rho	0.072	0.180	-0.005	-0.459 *	-0.406 *	—													
	p-value	0.715	0.359	0.978	0.014	0.032	—													
Confidence	Spearman's rho	-0.322	0.085	-0.024	-0.008	0.032	-0.024	—												
	p-value	0.094	0.667	0.902	0.967	0.870	0.905	—												
TMT B-A	Spearman's rho	-0.297	-0.330	-0.218	-0.344	-0.366	0.183	-0.207	—											
	p-value	0.159	0.116	0.306	0.099	0.079	0.391	0.332	—											
FMPS_PE	Spearman's rho	0.358	0.144	0.010	-0.030	-0.246	0.186	0.030	-0.191	—										
	p-value	0.079	0.491	0.964	0.888	0.236	0.373	0.888	0.382	—										
FMPS_DA	Spearman's rho	0.437 *	0.016	0.130	0.100	0.135	-0.051	-0.029	0.061	0.562 **	—									
	p-value	0.029	0.941	0.537	0.635	0.521	0.808	0.889	0.784	0.003	—									
FMPS_O	Spearman's rho	0.088	0.148	0.060	0.158	0.034	-0.018	-0.305	0.285	-0.225	0.195	—								
	p-value	0.677	0.480	0.776	0.450	0.873	0.931	0.139	0.188	0.280	0.349	—								
FMPS_SP	Spearman's rho	0.085	-0.010	-0.126	0.099	-0.212	-0.161	0.228	-0.259	0.607 **	0.473 *	-0.035	—							
	p-value	0.686	0.962	0.547	0.637	0.309	0.443	0.274	0.232	0.001	0.017	0.869	—							
SPSRQ_P	Spearman's rho	0.300	0.142	0.059	-0.082	-0.126	0.287	0.026	-0.057	0.535 **	0.596 **	0.177	0.353	—						
	p-value	0.121	0.473	0.767	0.678	0.524	0.139	0.894	0.791	0.006	0.002	0.397	0.083	—						
SPSRQ_R	Spearman's rho	0.013	0.013	0.006	0.011	-0.084	-0.050	0.591 ***	-0.108	0.268	0.229	-0.119	0.327	-0.014	—					
	p-value	0.946	0.947	0.977	0.956	0.670	0.799	<.001	0.616	0.196	0.271	0.572	0.111	0.945	—					
STAI_E	Spearman's rho	0.270	0.085	0.071	0.060	-0.110	0.356	0.041	0.210	0.335	0.193	-0.075	0.108	0.648 ***	-0.136	—				
	p-value	0.165	0.667	0.720	0.762	0.579	0.063	0.835	0.326	0.101	0.355	0.722	0.608	< .001	0.490	—				
STAI_T	Spearman's rho	0.361	-0.010	0.060	-0.042	-0.196	0.217	0.051	-0.129	0.742 ***	0.496 *	-0.093	0.460 *	0.765 ***	0.060	0.656 ***	—			
	p-value	0.059	0.960	0.762	0.831	0.317	0.266	0.797	0.549	< .001	0.012	0.659	0.021	< .001	0.761	< .001	—			
SSEI	Spearman's rho	-0.287	-0.023	-0.003	-0.003	0.032	-0.170	-0.131	-0.023	-0.364	-0.637 **	-0.215	-0.170	-0.835 ***	0.010	-0.561 **	-0.684 ***	—		
	p-value	0.164	0.913	0.988	0.987	0.880	0.417	0.533	0.918	0.087	0.001	0.325	0.438	< .001	0.961	0.004	< .001	—		
HAD_D	Spearman's rho	0.182	0.047	-0.126	0.098	-0.233	-0.050	0.065	0.139	0.468 *	0.334	0.064	0.326	0.412	-0.020	0.359	0.487 *	-0.302	—	
	p-value	0.407	0.832	0.565	0.658	0.286	0.820	0.767	0.547	0.032	0.140	0.782	0.149	0.051	0.927	0.093	0.019	0.173	—	
HAD_A	Spearman's rho	0.448 *	0.479 *	0.532 **	0.136	-0.102	0.201	-0.355	-0.132	0.567 **	0.368	0.124	0.188	0.589 **	-0.102	0.460 *	0.685 ***	-0.439 *	0.304	—
	p-value	0.032	0.021	0.009	0.537	0.645	0.359	0.097	0.569	0.007	0.101	0.593	0.415	0.003	0.642	0.027	< .001	0.041	0.158	—

Legend : * p < .05, ** p < .01, *** p < .001;

front_front : ΔFC between the orbital part of the left frontal inferior gyrus and the triangular part of the right frontal inferior gyrus ; **cere_cinguR/L** : ΔFC between the right cerebellar region 3 and right/left anterior cingulate cortices; **cere_tempo** : ΔFC between the right cerebellar region 3 and the right superior pole of the temporal lobe; **cere_front** : ΔFC between the right cerebellar region 3 and the orbital part of the right frontal inferior gyrus; **cauda_lingu** : ΔFC between the right caudate and left lingual gyrus
